# Temporal relationship between inflammation and insulin resistance and their joint effect on hyperglycemia: the Bogalusa Heart Study

**DOI:** 10.1186/s12933-019-0913-2

**Published:** 2019-08-23

**Authors:** Yinkun Yan, Shengxu Li, Yang Liu, Lydia Bazzano, Jiang He, Jie Mi, Wei Chen

**Affiliations:** 1 Department of Epidemiology, Capital Institute of Pediatrics, Beijing, China; 2 Department of Non-communicable Disease Management, Beijing Children’s Hospital, Capital Medical University, National Center for Children’s Health, Beijing, China; 3 Department of Epidemiology, Tulane University School of Public Health and Tropical Medicine, 1440 Canal Street, Room 1504G, New Orleans, LA USA; 4 Children’s Minnesota Research Institute, Children’s Hospitals and Clinics of Minnesota, Minneapolis, MN USA; 5 Department of Cardiology, The First Affiliated Hospital of Soochow University, Suzhou, China

**Keywords:** Inflammation, C-reactive protein, Insulin resistance, Diabetes

## Abstract

**Background:**

Inflammation and insulin resistance play crucial roles in the development of type 2 diabetes mellitus (T2DM). We aim to examine the temporal relationship between high-sensitivity C-reactive protein (hsCRP) and insulin resistance in non-diabetic adults and their joint effect on the development of hyperglycemia.

**Methods:**

The longitudinal cohort from the Bogalusa Heart Study consisted of 509 non-diabetic adults (360 whites and 149 blacks, mean age = 42.8 years at follow-up) who had hsCRP, fasting glucose and insulin measured twice at baseline and follow-up over 6.8 years. Cross-lagged panel model was used to examine the temporal relationship between hsCRP and homeostasis model assessment for insulin resistance (HOMA-IR). Information on incident T2DM was collected in a survey in 6.1 years after the follow-up survey.

**Results:**

After adjusting for race, sex, age, body mass index, smoking, alcohol drinking and follow-up years, the path coefficient from baseline hsCRP to follow-up HOMA-IR (β_2_ = 0.105, p = 0.009) was significant and greater than the path from baseline HOMA-IR to follow-up hsCRP (β_1_ = 0.005, p = 0.903), with p = 0.011 for the difference between β_1_ and β_2_. This one-directional path from baseline hsCRP to follow-up HOMA-IR was significant in the hyperglycemia group but not in the normoglycemia group. In addition, participants with high levels of baseline hsCRP and follow-up HOMA-IR had greater risks of T2DM (odds ratio, OR = 2.38, p = 0.035), pre-T2DM (OR = 2.27, p = 0.006) and hyperglycemia (OR = 2.18, p = 0.003) than those with low–low levels.

**Conclusions:**

These findings suggest that elevated hsCRP is associated with future insulin resistance in non-diabetic adults, and their joint effect is predictive of the development of T2DM.

**Electronic supplementary material:**

The online version of this article (10.1186/s12933-019-0913-2) contains supplementary material, which is available to authorized users.

## Background

Chronic inflammation has been shown to play a crucial role in the development of type 2 diabetes (T2DM) [1–4] and cardiovascular disease [5–9]. High-sensitivity C-reactive protein (hsCRP) is a sensitive marker of subclinical inflammation and strongly predicts increased risks of T2DM [10, 11]. Cross-sectional studies have shown that multiple inflammation markers including hsCRP are correlated with insulin resistance in diabetic and nondiabetic individuals [12, 13]. It is generally considered that hsCRP, insulin resistance, and T2DM are linked through obesity [14]. To date, there have been no studies focusing on the joint effect of increased hsCRP and insulin resistance on the development of T2DM independent of obesity.

The strong association between hsCRP and insulin resistance suggests that chronic inflammation and insulin resistance can influence each other based on pathophysiological and metabolic mechanisms [3, 15–17]. Inflammation may activate the innate immune system and interact with adipose tissue-specific macrophages [15, 17]. Although their relationship is likely bidirectional based on the existing literature in this regard, convincing evidence is lacking from the general population regarding the temporal relationship (causal sequence) between hsCRP and insulin resistance. Several longitudinal studies have demonstrated that higher baseline hsCRP and greater change in hsCRP are associated with the development of insulin resistance [18–21]; however, Mendelian randomization studies failed to demonstrate a causal relationship between hsCRP levels and insulin resistance/T2DM [22, 23]. Understanding the potential causal relationship between inflammation and insulin resistance may help provide new insights into the underlying mechanisms and yield novel approaches to prevent T2DM and cardiovascular diseases by focusing on interventions targeting at the causal factor.

Utilizing longitudinal data from the Bogalusa Heart Study, the current study aims to examine the temporal relationship between inflammation measured by hsCRP and homeostasis model assessment for insulin resistance (HOMA-IR) in non-diabetic adults and their joint effect on the development of T2DM.

## Methods

### Study population

The Bogalusa Heart Study is a series of long-term epidemiologic studies in a semi-rural, biracial (65% white and 35% black) community in Bogalusa, Louisiana founded by Dr. Gerald Berenson in 1973. This study focuses on the early natural history of cardiovascular disease from childhood [24]. Between 2000 and 2016, three cross-sectional surveys (baseline, follow-up and outcome surveys) of adults aged 24 to 58 years were conducted in Bogalusa, Louisiana. By linking the first two surveys (baseline in 2000–2002 and follow-up in 2006–2010), 755 non-diabetic adult participants were identified who had cardiovascular risk factors, including hsCRP, fasting plasma glucose and insulin, measured twice in both baseline and follow-up surveys, with an average follow-up period of 6.8 years. Among these 755 non-diabetic participants, 509 adults (360 Whites and 149 Blacks; 34.6% males, mean age = 49.3 years) were enrolled in the last survey (the outcome survey) in 2013–2016. These 509 participants who had incident T2DM and anti-diabetic medication information in the last survey formed the longitudinal cohort for the current study, with an average follow-up period of 6.1 years (range = 3.5–10.0 years) from the second (follow-up) survey.

All subjects gave informed consent for each survey. Study protocols were approved by the Institutional Review Board of the Tulane University Health Sciences Center.

### Measurements

Insulin, hsCRP, and glucose were measured at the same time at respective baseline and follow-up surveys. Standardized protocols were used by trained examiners across all surveys. Subjects were instructed to fast for 12 h before screening. Replicate measurements of height and weight were made, and the mean values were used for analysis. Body mass index (BMI, weight in kilograms divided by height in meters squared) was used as a measure of overall adiposity. Information on medication history, smoking and alcohol drinking was obtained in a questionnaire survey. Current smoking and alcohol drinking were defined as smoking at least one cigarette per day and consuming alcohol every day, respectively, during the prior 12 months.

Plasma hsCRP was measured by latex particle-enhanced immunoturbidimetric assay on Hitachi 902 Automatic Analyzer. Plasma glucose levels were measured as part of a multiple chemistry profile (SMA20; Laboratory Corporation of America, Burlington, NC). A commercial radioimmunoassay kit was used for measuring plasma immunoreactive insulin levels (Phadebas; Pharmacia Diagnostics, Piscataway, New Jersey). The intraclass correlation coefficients between blind duplicate values ranged from 0.86 to 0.98 for insulin, hsCRP, and glucose. HOMA-IR was calculated using the formula as described by Matthews [25]: HOMA-IR = fasting plasma insulin (μU/mL) × fasting plasma glucose (mmol/L)/22.5.

### Diagnosis of T2DM and pre-T2DM

T2DM was defined as fasting glucose ≥ 126 mg/dL or taking anti-diabetic medications. Prediabetes (pre-T2DM) was defined as fasting glucose of 100–125 mg/dL. Hyperglycemia was defined as being T2DM and/or pre-T2DM.

### Statistical analysis

Values for hsCRP, insulin, and HOMA-IR were log-transformed before subsequent analyses because of their skewed distributions. Analyses of covariance (generalized linear models) were performed to test differences in continuous study variables between race and sex groups.

**Fig. 1:**
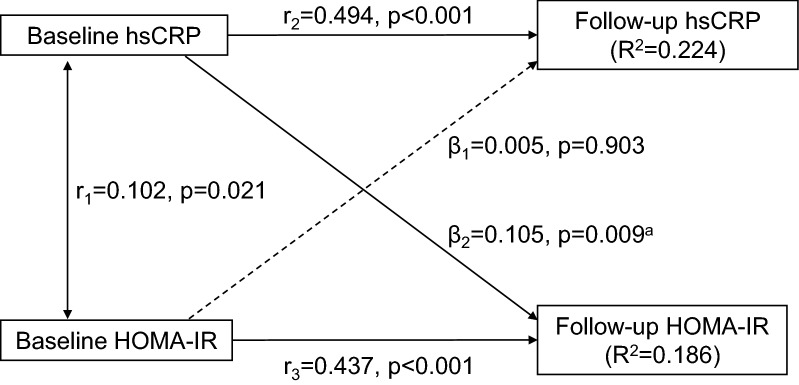
Cross-lagged path analysis models for hsCRP and HOMA-IR hsCRP, high-sensitivity C-reactive protein; HOMA-IR, homeostasis model assessment for insulin resistance; β, standardized regression coefficient; r_1_, synchronous correlation; r_2_ and r_3_, tracking correlations. Goodness-of-fit: root mean square residual = 0.037 and comparative fit index = 0.960. a, p = 0.011 for the difference between β_1_ and β_2_

The study design of longitudinal changes of hsCRP, glucose, and insulin measured at two time points in the baseline and follow-up surveys was typically a cross-lagged panel design [26, 27]. The cross-lagged panel analysis is a form of path analysis that simultaneously examines reciprocal and longitudinal relationships among a set of intercorrelated variables. Kenny et al. first proposed the cross-lagged analysis model by calculating partial correlation coefficients to measure the direct paths [26]. In more recent years, structural equation modeling has been used to estimate the path coefficients [27]. A simplified and conceptual version of the model is presented in Fig. 1. The path with β_1_ describes the effect of baseline HOMA-IR on subsequent hsCRP, and the path with β_2_ describes the effect of baseline hsCRP on subsequent HOMA-IR. Prior to cross-lagged path analysis, the baseline and follow-up values of log-transformed hsCRP and log-transformed HOMA-IR were adjusted for age, BMI, smoking, and alcohol drinking by regression residual analyses and then standardized by Z-transformation (mean = 0, SD = 1) in race-sex groups. The four variables, i.e. hsCRP and HOMA-IR at baseline and follow-up, generated six pair-wise observed correlations, and five correlations in Fig. 1 were used to estimate β_1_ and β_2_ because the connecting path through hsCRP-HOMA-IR correlation at follow-up is illegal according to the path analysis rules. Pearson correlation coefficients of the covariate-adjusted and Z-transformed variables of hsCRP and HOMA-IR at baseline and follow-up were calculated; correlation coefficients between baseline and follow-up values were calculated with additional adjustment for follow-up years. The cross-lagged path coefficients (β_1_ and β_2_) in the path diagram in Fig. 1 were estimated simultaneously based on the correlation matrix using the maximum likelihood method by the program LISREL 8.52. The validity of model fitting was indicated by root mean square residual (RMR) and comparative fit index (CFI) [28]. A significant path coefficient (β_1_ or β_2_) suggests the directionality, and a significant difference between β_1_ and β_2_ provides stronger evidence for the directional path between the two variables measured over time.

Stratified analyses were performed to examine the difference in cross-lagged path parameters between groups classified by glycemic status, race, sex, and follow-up period. The difference between β_1_ and β_2_ derived from the standardized variables (Z-scores) was tested using Fisher’s Z-test. Multivariable logistic regression models were used to examine the joint effect of baseline hsCRP and follow-up HOMA-IR on T2DM, pre-T2DM, and hyperglycemia. Low and high levels of baseline hsCRP and follow-up HOMA-IR were defined by their medians.

## Results

### Characteristics of participants

The mean levels of continuous variables were compared between race and sex groups, adjusting for age (except age itself). Although log-transformed hsCRP, insulin, and HOMA-IR were used for subsequent analyses, the medians (interquartile ranges) of their original values were presented in Table 1. Race differences in BMI, insulin, and HOMA-IR (blacks > whites) in females and sex differences in glucose (males > females), insulin (males > females), HOMA-IR (males > females) and hsCRP (males < females) in whites were observed in both baseline and follow-up surveys. In the outcome survey, race differences in BMI (blacks > whites) in females and incidence of T2DM (blacks > whites) in males and sex differences in hyperglycemia (males > females) and pre-T2DM (males > females) in blacks and whites were found.

**Table 1:** Characteristics of study participants in baseline, follow-up and outcome surveys by race and sex

	White		Black		p for race difference^a^	
	Male (n = 138)	Female (n = 222)	Male (n = 38)	Female (n = 111)	Male	Female
Baseline survey						
Age (years)	36.4 (4.5)	36.2 (4.2)	36.6 (4.2)	35.0 (4.5)	0.975	0.041
BMI (kg/m^2^)	28.1 (4.9)	27.2 (6.2)	29.4 (6.7)	31.3 (7.8)	0.286	< 0.001
Smoking, n (%)	35 (25.4)	41 (18.5)	15 (39.5)	33 (29.7)	0.088	0.020
Alcohol drinking, n (%)	54 (39.1)	98 (44.1)	8 (21.1)	42 (37.8)	0.039	0.272
Glucose (mg/dL)	83.5 (9.4)**	79.6 (8.1)	83.2 (8.9)	81.2 (9.3)	0.861	0.121
Insulin (μU/mL)^b^	10.0 (7.0–14.0)*	8.0 (6.0–13.0)	9.0 (5.0–17.0)	11.0 (7.0–17.0)	0.782	0.001
HOMA-IR^b^	2.04 (1.45–2.82)*	1.65 (1.11–2.60)	1.89 (1.06–3.66)	2.27 (1.43–3.46)	0.786	0.001
hsCRP (mg/L)^b^	0.93 (0.37–1.93)**	1.65 (0.71–4.15)	1.78 (0.60–3.09)	2.07 (0.58–4.80)	0.131	0.830
Pre-diabetes, n (%)	5 (3.6)	3 (1.4)	2 (5.3)	2 (1.8)	0.155	0.255
Follow-up survey						
Age (years)	43.2 (4.7)	43.0 (4.5)	43.6 (4.5)	41.9 (4.7)	0.674	0.043
Follow-up period (years)	6.8 (0.9)	6.8 (0.9)	7.0 (0.8)	6.9 (0.9)	0.222	0.123
BMI (kg/m^2^)	29.6 (5.6)	28.6 (6.7)	31.8 (8.2)	33.0 (8.2)	0.131	< 0.001
Smoking, n (%)	32 (23.2)	50 (22.5)	15 (39.5)	33 (29.7)	0.045	0.152
Alcohol drinking, n (%)	52 (37.7)	91 (41.0)	7 (18.4)	38 (34.2)	0.026	0.233
Glucose (mg/dL)	90.6 (10.7)**	86.7 (8.8)	92.5 (12.2)**	88.8 (8.6)	0.345	0.036
Insulin (μU/mL)^b^	10.1 (6.3–16.2)*	8.2 (5.1–13.0)	13.6 (6.7–24.0)	12.8 (6.96–18.9)	0.052	< 0.001
HOMA-IR^b^	2.26 (1.35–3.63)*	1.74 (1.04–3.00)	3.33 (1.20–5.78)	2.82 (1.43–4.56)	0.065	< 0.001
hsCRP (mg/L)^b^	0.93 (0.41–1.97)**	1.35 (0.52–3.14)	1.09 (0.56–2.94)	2.07 (0.58–4.80)	0.186	0.062
Pre-diabetes, n (%)	22 (15.9)*	19 (8.6)	11 (29.0)**	10 (9.0)	0.069	0.891
Outcome survey						
Age (years)	49.6 (4.5)	49.5 (4.2)	50.1 (4.2)*	48.0 (4.7)	0.547	0.003
Follow-up period (year)^c^	6.0 (1.1)	6.1 (1.1)	6.2 (1.1)	5.9 (1.1)	0.386	0.067
BMI (kg/m^2^)	30.3 (5.4)	29.5 (6.7)	32.3 (8.6)	34.9 (8.5)	0.085	< 0.001
Smoking, n (%)	33 (23.9)	45 (20.3)	20 (52.6)**	27 (24.3)	< 0.001	0.397
Alcohol drinking, n (%)	71 (51.5)	88 (55.4)	18 (47.4)**	18 (16.2)	0.656	< 0.001
Hyperglycemia, n (%)	76 (55.1)**	78 (35.1)	27 (71.1)*	50 (45.1)	0.077	0.080
Pre-diabetes, n (%)	61 (44.2)**	51 (22.9)	17 (44.8)*	34 (30.7)	0.288	0.091
T2DM, n (%)	15 (10.9)	27 (12.2)	10 (26.3)	16 (14.4)	0.016	0.563

Continuous variables are presented as means (SD) or medians (interquartile range)

BMI, body mass index; hsCRP, high-sensitivity C-reactive protein; HOMA-IR, homeostasis model assessment for insulin resistance; T2DM, type 2 diabetes mellitus

Sex difference within race: * p < 0.05; ** p < 0.01

^a^p values for race difference in continuous variables were adjusted for age

^b^Median (interquartile range)

^c^Follow-up period from the time point of the follow-up survey

### Correlations between hsCRP and HOMA-IR at baseline and follow-up

After adjusting for race, sex, age, BMI, smoking and alcohol use, with additional adjustment for follow-up years for baseline-follow-up correlations, most correlation coefficients between baseline and follow-up values of log-hsCRP and log-HOMA-IR were significant in the total sample and subgroups based on the cut-off values for the significance level (p < 0.05) listed below the table. All the coefficients did not differ between subgroups except for the difference in coefficients of follow-up log-hsCRP and log-HOMA-IR between races (0.248 vs 0.043) (Table 2).

**Table 2:** Pearson correlation coefficients between log-transformed hsCRP and HOMA-IR at baseline and follow-up in the total cohort and subgroups, adjusted for covariates

Group	Variable	Baseline hsCRP	Baseline HOMA-IR	Follow-up hsCRP
Total^a^	Baseline HOMA-IR	0.102		
	Follow-up hsCRP	0.473	0.053	
	Follow-up HOMA-IR	0.147	0.418	0.187
White/black^b^	Baseline HOMA-IR	0.108/0.090		
	Follow-up hsCRP	0.494/0.416	0.082/− 0.014	
	Follow-up HOMA-IR	0.154/0.152	0.437/0.369	0.248/0.043*
Male/female^c^	Baseline HOMA-IR	0.036/0.139		
	Follow-up hsCRP	0.444/0.495	0.029/0.068	
	Follow-up HOMA-IR	0.060/0.190	0.460/0.396	0.161/0.201
Normo/hyper^d^	Baseline HOMA-IR	0.146/0.043		
	Follow-up hsCRP	0.483/0.455	0.034/0.064	
	Follow-up HOMA-IR	0.129/0.175	0.340/0.437	0.121/0.268

Covariates included in the models were race, sex, age, BMI, smoking and alcohol use for the total sample and Normo/Hyper groups, with additional adjustment for follow-up years for baseline-follow-up correlations

hsCRP, high-sensitivity C-reactive protein; HOMA-IR, homeostasis model assessment for insulin resistance; Normo, normoglycemia in the outcome survey; Hyper, hyperglycemia in the outcome survey

^a^Correlation coefficients greater than 0.087 are significant (p < 0.05)

^b^Correlation coefficients greater than 0.103 for whites and 0.161 for blacks are significant (p < 0.05)

^c^Correlation coefficients greater than 0.148 for males, and 0.108 for females are significant (p < 0.05)

^d^Correlation coefficients greater than 0.118 for normoglycemia, and 0.129 for hyperglycemia are significant (p < 0.05)

* p < 0.05 for race difference

### Cross-lagged path analysis of hsCRP and HOMA-IR

After adjusting for race, sex, age, BMI, smoking, alcohol drinking, and follow-up years, the path coefficient from baseline hsCRP to follow-up HOMA-IR (β_2_ = 0.105, p = 0.009) was greater than the path coefficient from baseline HOMA-IR to follow-up hsCRP (β_1_ = 0.005, p = 0.903), with p = 0.011 for the difference between β_1_ and β_2_. The tracking correlations of hsCRP and HOMA-IR from baseline to follow-up and the synchronous correlation between hsCRP and HOMA-IR at baseline were significant. The variance (R^2^) of follow-up hsCRP (0.224) and follow-up HOMA-IR (0.186) explained by baseline predictors was significant. RMR and CFI were 0.037 and 0.960, respectively, indicating a good fit to the observed data according to the criteria of RMR < 0.05 and CFI > 0.90 (Fig. 1).

### Cross-lagged path analyses by subgroups

The path coefficients (β_1_) of baseline HOMA-IR → follow-up hsCRP in all subgroups were nonsignificant (p > 0.05). The path coefficients (β_2_) of baseline hsCRP → follow-up HOMA-IR in subgroups of hyperglycemia (β_2_ = 0.156, p = 0.008), pre-T2DM (β_2_ = 0.177, p = 0.012), whites (β_2_ = 0.108, p = 0.023), females (β_2_ = 0.138, p = 0.006) and follow-up years above median (β_2_ = 0.199, p < 0.001) were significant. Significant differences in the path coefficients (β_1_ and β_2_) were noted only between normoglycemia and T2DM groups and between follow-up period groups. The model fitting parameters of RMR ranged from 0.002 to 0.148 and CFI from 0.862 to 1.000, indicating a relatively good fit to the observed data (Table 3). The path coefficient of baseline hsCRP → follow-up insulin (β_2_ = 0.107, p = 0.008) was greater than the path of baseline insulin → follow-up hsCRP (β_1_ = − 0.001, p = 0.998), with p = 0.004 for the difference between β_1_ and β_2_ (Additional file 1: Fig. S1). In contrast, neither the path coefficient of hsCRP → glucose (β_2_ = 0.040, p = 0.339) nor the path of glucose → hsCRP (β_1_ = − 0.025, p = 0.520) was significant. In addition, the path coefficient of hsCRP → insulin (β_2_ = 0.152, p = 0.009) was significant in the hyperglycemia group, but not significant in the normoglycemia group (β_2_ = 0.088, p = 0.121) (Additional file 1: Fig. S2).

**Table 3:** Cross-lagged path coefficients between log-transformed hsCRP and HOMA-IR by glycemia status, race, sex, and follow-up period

	Path coefficients					Goodness-of-fit	
	HOMA-IR → hsCRP		hsCRP → HOMA-IR		p^a^	RMR	CFI
	β_1_	p	β_2_	p			
Glycemia status							
Normoglycemia (n = 278)	− 0.037	0.483	0.081	0.155	0.032	0.022	0.990
Hyperglycemia (n = 231)	0.045	0.449	0.156	0.008	0.059	0.054	0.914
T2DM (n = 68)	0.131	0.206	0.143	0.199	0.911	0.031	0.994
Pre-T2DM (n = 163)	0.006	0.938	0.177	0.012	0.015	0.061	0.884
p^b^	0.141		0.194				
p^c^	0.011		0.380				
p^d^	0.475		0.121				
Race							
White (n = 360)	0.029	0.530	0.108	0.023	0.111	0.097	0.932
Black (n = 149)	− 0.052	0.490	0.120	0.117	0.022	0.148	1.000
p^e^	0.148		0.843				
Sex							
Male (n = 176)	0.013	0.848	0.044	0.518	0.645	0.104	0.928
Female (n = 333)	− 0.001	0.986	0.138	0.006	0.005	0.111	0.962
p^f^	0.801		0.095				
Follow-up period							
Below median (n = 245)	− 0.018	0.756	0.035	0.560	0.352	0.067	0.862
Above median (n = 264)	0.026	0.627	0.199	< 0.001	< 0.001	0.002	0.981
p^g^	0.885		0.003				

β, standardized regression coefficient; RMR, root mean square residual; CFI, comparative fit index; hsCRP, high-sensitivity C-reactive protein; HOMA-IR, homeostasis model assessment for insulin resistance; T2DM, type 2 diabetes mellitus

^a^p value for difference between β_1_ and β_2_

^b^p value for difference in β_1_ and β_2_ between hyperglycemia and normoglycemia groups

^c^p value for difference in β_1_ and β_2_ between T2DM and normoglycemia groups

^d^p value for difference in β_1_ and β_2_ between pre-T2DM and normoglycemia groups

^e^p value for difference in β_1_ and β_2_ between whites and blacks

^f^p value for difference in β_1_ and β_2_ between males and females

^g^p value for difference in β_1_ and β_2_ between follow-up period groups (median = 6.7 years)

### Joint effect of inflammation and insulin resistance on hyperglycemia

**Fig. 2:**
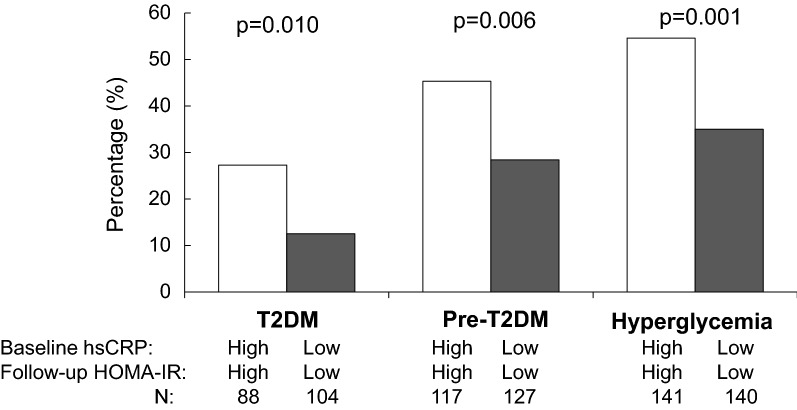
Percentage of T2DM, Pre-T2DM and hyperglycemia in the outcome survey between high–high and low–low groups of baseline hsCRP and follow-up HOMA-IR. hsCRP, high-sensitivity C-reactive protein; HOMA-IR, homeostasis model assessment for insulin resistance; T2DM, type 2 diabetes mellitus. High and low levels of hsCRP and HOMA-IR were defined by their medians

The percentages of T2DM (p = 0.010), pre-T2DM (p = 0.006) and hyperglycemia (p = 0.001) in the outcome survey were significantly greater among participants with high hsCRP at baseline and high HOMA-IR at follow-up (high–high group) than among participants with low hsCRP at baseline and low HOMA-IR at follow-up (low–low group) (Fig. 2). After adjusting for race, sex, age, BMI, smoking, and alcohol drinking, participants in the high–high group had greater risks of T2DM (OR = 2.38, 95% CI 1.06–5.53), pre-T2DM (OR = 2.27, 95% CI 1.27–4.05) and hyperglycemia in the outcome survey (OR = 2.18, 95% CI 1.30–3.64) than those in the low–low group (Table 4).

**Table 4:** ORs for T2DM, Pre-T2DM, and hyperglycemia in the outcome survey associated with the high–high group with the low–low group as reference, adjusting for covariates

Independent variable	Outcome					
	T2DM (37 vs 155)		Pre-T2DM (89 vs 155)		Hyperglycemia (126 vs 155)	
	OR (95% CI)	p	OR (95% CI)	p	OR (95% CI)	p
Black race	1.14 (0.50–2.63)	0.758	0.98 (0.51–1.90)	0.953	1.07 (0.60–1.91)	0.823
Female sex	0.72 (0.30–1.73)	0.467	0.39 (0.21–0.72)	0.003	0.49 (0.28–0.84)	0.010
Age	1.05 (0.96–1.16)	0.286	1.10 (1.02–1.17)	0.010	1.08 (1.02–1.15)	0.011
BMI	1.07 (1.01–1.12)	0.014	1.09 (1.05–1.14)	< 0.001	1.08 (1.04–1.12)	< 0.001
Smoking	1.74 (0.71–4.25)	0.224	1.17 (0.59–2.32)	0.647	1.28 (0.70–2.34)	0.419
Alcohol drinking	0.30 (0.11–0.86)	0.025	1.06 (0.57–1.96)	0.855	0.77 (0.44–1.36)	0.367
Group^a^	2.38 (1.06–5.33)	0.035	2.27 (1.27–4.05)	0.006	2.18 (1.30–3.64)	0.003

High and low levels of baseline hsCRP and follow-up HOMA-IR were defined by their medians

OR, odds ratio; CI, confidence interval; T2DM, type 2 diabetes mellitus; BMI, body mass index; hsCRP, high-sensitivity C-reactive protein; HOMA-IR, homeostasis model assessment for insulin resistance

^a^Coding: high–high group = 1; low–low group = 0

## Discussion

### Principal findings

This community-based longitudinal cohort study of black and white adults examined the temporal relationship between inflammation measured by hsCRP and insulin resistance estimated by HOMA-IR in a community-based cohort using a cross-lagged path analysis model, a statistical approach to dissecting a causal relationship between inter-correlated variables [26, 27]. We found that higher hsCRP preceded HOMA-IR rather than vice versa in non-diabetic adults, and this one-directional relationship was found to be significant in pre-T2DM and hyperglycemia groups but not in the normoglycemia group; high levels of baseline hsCRP and follow-up HOMA-IR were significantly associated with increased risks of T2DM, pre-T2DM and hyperglycemia. The findings suggest that hsCRP levels are associated with subsequent HOMA-IR in non-diabetic adults, and their joint effect predicts the development of T2DM.

### Temporal relationship between inflammation and insulin resistance

Inflammation and insulin resistance are two well-known mechanisms linking obesity, T2DM and cardiovascular disease [29, 30]. Multiple inflammation markers including hsCRP are correlated with hyperglycemia and insulin resistance in majority of previous studies [13, 21, 31–36]. An important question raised by these observations is whether adiposity-induced inflammation reaction precedes insulin resistance or vice versa, or whether this relationship is bidirectional, especially in the evolution of T2DM. Cross-sectional study design limits the inference regarding the temporal relationship between hsCRP and insulin resistance. Several longitudinal studies have attempted to demonstrate the temporal relationship between inflammation, insulin resistance, and T2DM [10, 11, 18, 19]. Multiple inflammatory markers, including hsCRP, interleukin-6, and plasminogen activator inhibitor-1, have been shown to independently predict incident T2DM [10, 11]. The Coronary Artery Risk Development in Young Adults study showed that elevated hsCRP was associated with concurrent and future insulin resistance after accounting for adiposity and oxidative stress markers [18]. To date, only one longitudinal study has examined the bidirectional relationship between inflammation and insulin resistance and showed that baseline levels of hsCRP and interleukin-6 were positively associated with subsequent increases in fasting insulin, HOMA-IR, and beta-cell function. In the reverse analysis, baseline HOMA-IR was not associated with change in inflammation biomarkers [19]. However, these traditional longitudinal analysis models cannot address the causal relationship. The present study confirmed and extended previous findings by showing that higher hsCRP preceded insulin resistance among non-diabetic adults using a cross-lagged path analysis model. This one-directional temporal relationship was observed only in participants who had hyperglycemia but not in normoglycemic adults. In addition to HOMA-IR, we found that hsCRP elevation preceded increased fasting insulin but not for fasting glucose, suggesting that insulin levels contribute more than glucose to the HOMA-IR index to measure insulin resistance, and fasting insulin alone may be a simple and effective surrogate measure of insulin resistance [37]. The causal relationship between hsCRP and insulin resistance may account for the observation that anti-inflammatory treatments can improve glycemia and insulin resistance among diabetic patients [38, 39].

The mechanisms linking inflammation to insulin resistance are incompletely elucidated. Existing data support the concept that the temporal relationship between chronic inflammation and insulin resistance is bidirectional like a “two-way street”, i.e. they are mutually influenced based on the underlying metabolic and physiologic mechanisms [3, 15]. Previous studies suggest that inflammation may promote insulin resistance through the production of proinflammatory cytokines such as interleukin-1β, interacting with adipose tissue-specific macrophages and activation of innate immune system [3, 15, 40]. In addition, inhibition of inflammatory signaling by knockout of key pathways in obese mice can directly prevent the development of insulin resistance [41, 42]. On the other hand, experimental studies in mice have suggested that insulin resistance may cause inflammation through the production of the chemokine monocyte protein 1 [16]. It is well-known that inflammation and insulin resistance are linked through obesity [14]. In the present observational population study, the results from the cross-lagged analyses indicated that elevated hsCRP occurred prior to increased HOMA-IR independent of BMI in non-diabetic participants, but the reverse was not true. The finding of this one-directional time sequence from our study provided a necessary first step in establishing causation between systemic inflammation and insulin resistance. Further large and well-controlled prospective clinical trials targeting inflammatory pathways for its treatment are warranted [43].

Several studies have reported significant sex and black-white differences in the cross-sectional association between hsCRP and HOMA-IR [44–46]. In the present study, however, we did not find significant sex and race differences in the cross-sectional correlations (except for the correlation at follow-up) and longitudinal directional path parameters between hsCRP and HOMA-IR. These inconsistencies may be due to limited sample size and different age periods, and thus future studies with larger sample sizes are needed to assess the sex- and race-specific association, particularly in the temporal relationship analysis.

### Joint effect of inflammation and insulin resistance on T2DM

Previous longitudinal studies have demonstrated independent effects of hsCRP and HOMA-IR in predicting the risk of T2DM [47, 48]; however, their joint effect on the development of T2DM has not been reported. In the present study, two approaches were applied to test the joint effect of hsCRP and HOMA-IR on T2DM, pre-T2DM and hyperglycemia, i.e. estimating the effect of baseline hsCRP on follow-up HOMA-IR in glycemic groups and assessing the risk of hyperglycemia of the group with high levels of baseline hsCRP and follow-up HOMA-IR with the low–low group as reference. We found that the path coefficients of baseline hsCRP → follow-up HOMA-IR were significant in the pre-T2DM and hyperglycemia groups; individuals with high levels of baseline hsCRP and follow-up HOMA-IR had significantly increased risks of T2DM, pre-T2DM and hyperglycemia compared with those who had low–low levels. The results suggest that inflammation and insulin resistance have a joint effect on the pathogenesis of hyperglycemia.

### Strengths and limitations

This community-based longitudinal cohort study provides a unique opportunity to examine the temporal relationship between increased hsCRP and insulin resistance and their joint effect on hyperglycemia. However, several limitations should be noted. First, the relatively small sample size of subgroups, especially the number of T2DM patients, had a limited statistical power to detect a weak-to-moderate association. Second, we assessed chronic inflammation state using hsCRP, which is a downstream inflammatory marker. Further studies are required involving a wider spectrum of inflammatory biomarkers such as fibrinogen, interleukin 6, and tumor necrosis factor-α. Third, T2DM was diagnosed based on fasting blood glucose and anti-diabetic medication history in this study without 2 h blood glucose data, which may lead to missed diagnosis.

## Conclusions

This study demonstrates that elevated CRP is associated with future insulin resistance in non-diabetic adults, and their joint effect is predictive of the development of T2DM. The findings will improve our understanding of the pathobiology, mechanisms, and natural history of T2DM. Interventional studies are warranted regarding whether anti-inflammatory therapy is an effective strategy for reducing the risk of insulin resistance and T2DM.

## Additional file

**Additional file 1.** Additional figures.

## Data Availability

All authors had full access to all of the data in the study and take responsibility for the integrity of the data and the accuracy of the data analysis. The datasets used and/or analyzed during the current study are available from the corresponding author on reasonable request.

## Abbreviations

T2DM: type 2 diabetes
hsCRP: high-sensitivity C-reactive protein
HOMA-IR: homeostatic model assessment for insulin resistance
RMR: root mean square residual
CFI: comparative fit index
OR: odds ratio
CI: confidence interval
