# Supplementary material for: Temporal relationship between inflammation and insulin resistance and their joint effect on hyperglycemia: the Bogalusa Heart Study
Source: Cardiovasc Diabetol. 2019 Aug 23;18:109. doi: 10.1186/s12933-019-0913-2 (PMC6706925; doi:10.1186/s12933-019-0913-2)

**Online-Only Additional Material**

**Temporal Relationship between Inflammation and Insulin Resistance and Their Joint Effect on Hyperglycemia: the Bogalusa Heart Study**

Yinkun Yan^1,2^, MD, PhD, Shengxu Li^2^, MD, PhD, Yang Liu^2,3^, MD, PhD, Lydia Bazzano^2^, MD, PhD, Jiang He^2^, MD, PhD, Jie Mi^1^, MD, PhD, Wei Chen^2^, MD, PhD

^1^ Beijing Children’s Hospital, Capital Medical University, National Center for Children’s Health, Beijing, China

^2^ Department of Epidemiology, Tulane University School of Public Health and Tropical Medicine, New Orleans, LA

^3^ Department of Cardiology, The First Affiliated Hospital of Soochow University, Suzhou, China

**Short title:** Inflammation, Insulin Resistance and Diabetes

**Correspondence to:**

Wei Chen, MD, PhD,

1440 Canal Street, Room 1504G,

New Orleans, LA 70112.

Tel: (504) 988-7611; Fax: (504) 988-7194

Email: wchen1@tulane.edu

The authors do not have any conflict of interest.


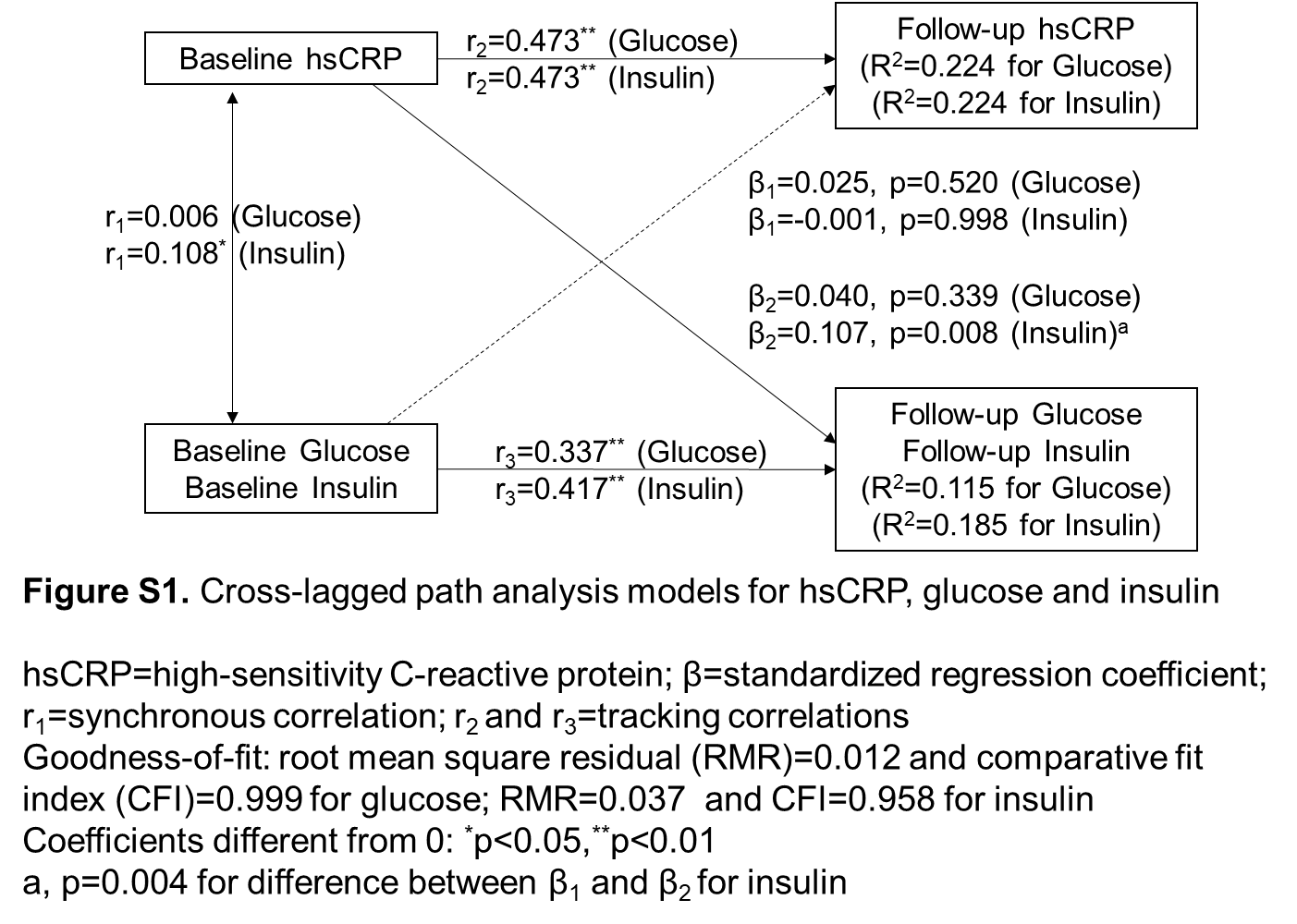


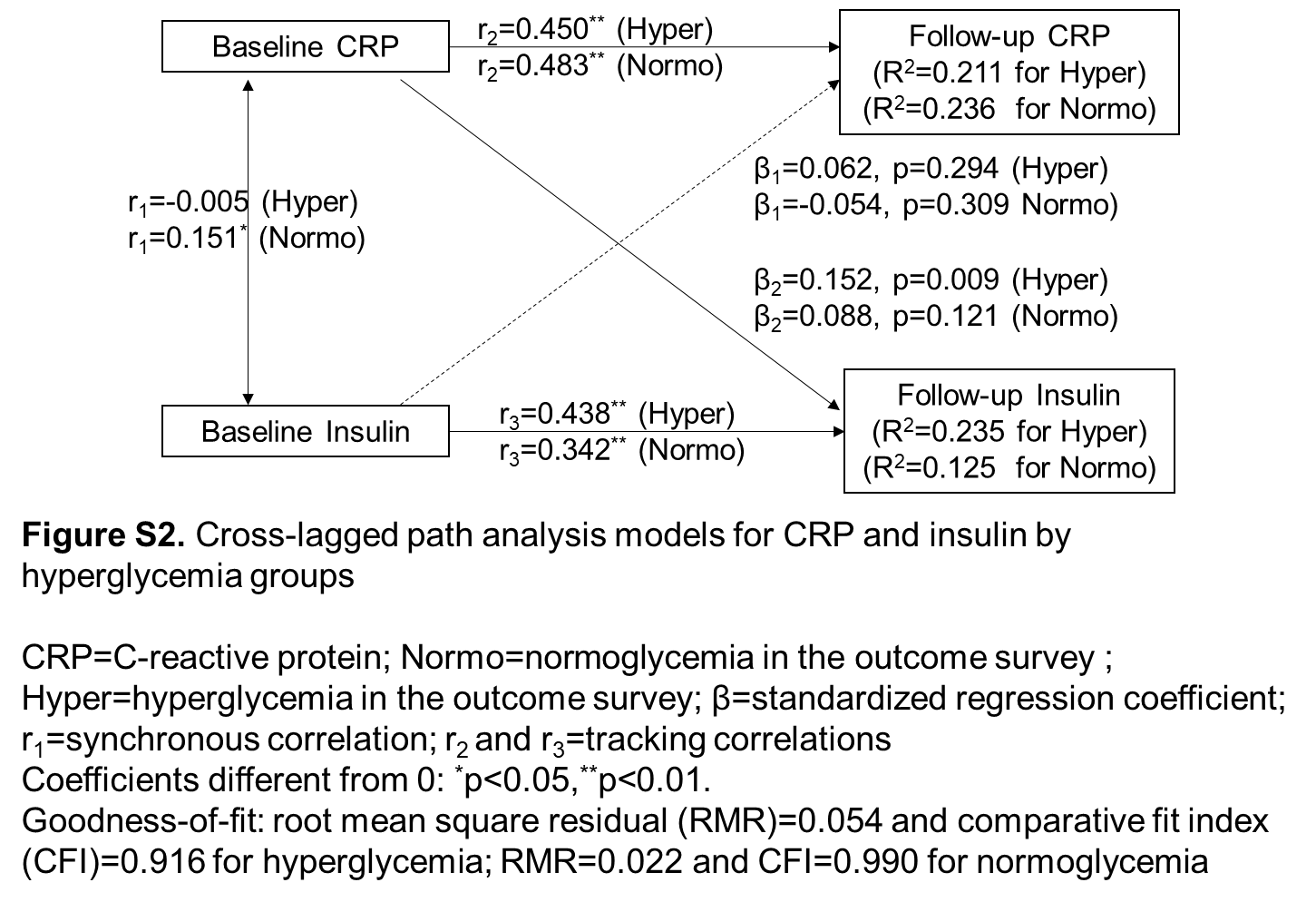

Supplement: Supplementary file 1 — Additional file 1. Additional figures. [file 12933_2019_913_MOESM1_ESM.docx]
